# The Relationship Between Holistic Humanistic Caring Perception, Thriving at Work and Clinical Nursing Competency Among Newly Graduated Nurses: A Mediation Study With Latent Profile Analysis

**DOI:** 10.1155/jonm/1356643

**Published:** 2026-10-21

**Authors:** Yuping Zhang, Guimei Wang, Weihua Hou, Dandan Shi, Xiaobing Yin

**Affiliations:** ^1^ Department of Medical Affairs Shanghai Tenth People’s Hospital Tongji University School of Medicine Shanghai 200072 China tongji.edu.cn; ^2^ School of Nursing and Health Henan University Kaifeng 475000 China henu.edu.cn; ^3^ Department of Nursing Shanghai Tenth People’s Hospital Tongji University School of Medicine Shanghai 200072 China tongji.edu.cn

**Keywords:** clinical nursing competency, holistic humanistic caring perception, latent profile analysis, newly graduated nurses, thriving at work

## Abstract

**Aim:**

To identify latent profiles of holistic humanistic caring perception among newly graduated nurses, examine associated factors and assess indirect associations with clinical nursing competency through thriving at work.

**Background:**

Heterogeneity in caring perception and the role of thriving at work in clinical nursing competency remain unclear.

**Methods:**

A cross‐sectional study included 421 newly graduated nurses from six tertiary hospitals. Latent profile analysis, multinomial logistic regression and covariate‐adjusted mediation analysis with 5000 bootstrap samples were performed.

**Results:**

Three profiles characterised mainly by low (30.9%), moderate (31.8%) and high (37.3%) caring perception were identified. Only‐child status, living situation and work experience were associated with profile membership. Significant indirect associations through thriving at work were found for high versus low (*B* = 46.17, 95% bootstrap CI 36.73–55.58) and moderate versus low (*B* = 27.26, 95% bootstrap CI 21.30–33.45). The direct association remained significant for high versus low but not moderate versus low.

**Conclusions:**

Caring profiles were associated with clinical nursing competency, with significant indirect associations through thriving at work.

**Implications for Nursing Management:**

Profile‐specific caring support and conditions that foster learning and vitality may help nursing managers support competency development.

## 1. Introduction

Nurses constitute a core workforce in healthcare systems and play an important role in the delivery of safe and high‐quality care [1–3]. Newly graduated nurses represent an important group entering the nursing workforce. In this study, newly graduated nurses were defined as nurses who had graduated from nursing school and entered professional nursing practice within the previous 2 years [4]. This period represents a critical transition from student to practitioner and from theoretical learning to clinical practice [5], during which the development of clinical nursing competency is important for ensuring the quality and safety of nursing care [6].

Clinical nursing competency is a holistic and integrated capacity that enables nurses to fulfil their professional responsibilities effectively in clinical practice [7]. In this study, clinical nursing competency was assessed using the Competency Scale for Clinical Nurses, which comprises five domains: knowledge, professional skills, personal traits, motivation and social competence [8]. Newly graduated nurses may experience gaps in clinical nursing competency during the transition to independent practice [9]. Previous studies have reported deficiencies in medication management, direct clinical practice and communication among newly graduated nurses. Such deficiencies may increase the risk of medication errors and adverse events and compromise nurse–patient interactions and the quality of care [10–12]. Therefore, identifying factors associated with clinical nursing competency among newly graduated nurses is important for supporting their competency development and professional growth.

Holistic humanistic caring perception refers to nurses’ perceived caring experiences from six sources: self, hospital management, the department, patients and their families, family members and friends or peers [13]. Given that these sources span personal, work and nonwork contexts, conservation of resources theory may provide a useful framework for understanding their relevance to newly graduated nurses’ professional adaptation [14]. Conservation of resources theory proposes that individuals strive to obtain, retain, foster and protect resources they value [14]. It further suggests that individuals with greater resources are better positioned to acquire additional resources, such that resource gains may facilitate further gains [15]. Newly graduated nurses face substantial demands while acquiring clinical experience and adapting to independent practice, which may deplete their available psychological and social resources. From a conservation of resources perspective, caring perceived across different contexts can be conceptualised as resources available to newly graduated nurses. Within this framework, self‐care was conceptualised as a resource arising from the personal domain; caring from hospital management, the department and patients and their families as work‐related resources; and caring from family and friends as nonwork social resources. Although these resources arise from different contexts, they may operate in complementary ways to support learning, adaptation and sustained engagement in clinical practice. Together, these processes may contribute to the development of clinical nursing competency. Greater holistic humanistic caring perception may therefore be associated with higher clinical nursing competency among newly graduated nurses. Previous research has shown that perceived humanistic caring is associated with nurses’ work engagement [16]. Among newly graduated nurses, organisational support and family functioning have also been associated with reality shock during the transition to clinical practice [17]. Together, these findings support further consideration of holistic humanistic caring perception in newly graduated nurses, although its association with clinical nursing competency remains unclear.

Thriving at work may represent an important psychological process linking holistic humanistic caring perception with clinical nursing competency. Thriving at work is a positive psychological state characterised by learning and vitality [18]. Learning reflects the acquisition and refinement of professional knowledge and skills, whereas vitality reflects the energy available to remain actively engaged in demanding clinical practice. These two components are therefore particularly relevant to clinical nursing competency development among newly graduated nurses. Previous studies have shown that higher levels of thriving at work are associated with better job performance, lower work stress and lower turnover intention among nurses [19–21]. Supportive work environments have also been associated with thriving among early‐career nurses [22]. From a conservation of resources perspective [14], the caring reflected in holistic humanistic caring perception can provide newly graduated nurses with resources to cope with the demands of transition to clinical practice. Greater availability of these resources may facilitate positive psychological states such as thriving at work. Learning and vitality, the two components of thriving at work, are also relevant to the accumulation of clinical knowledge and skills, sustained engagement in practice and adaptation to professional roles. Thriving at work may therefore link holistic humanistic caring perception with clinical nursing competency.

Variable‐centred approaches are useful for examining average associations between variables but do not directly identify subgroups of individuals characterised by different joint patterns across multiple dimensions. This limitation is particularly relevant to holistic humanistic caring perception, which comprises caring perceived from six different sources. Analyses based on an overall score may obscure differences in the relative pattern of caring across these sources. In contrast, latent profile analysis (LPA) provides a person‐centred approach to identifying subgroups of individuals who share similar multidimensional response patterns [23]. LPA may therefore provide a more nuanced understanding of heterogeneity in holistic humanistic caring perception by identifying subgroups based on the joint patterns of caring across different sources, which may not be captured by the overall score alone.

Accordingly, this study aimed to: (1) identify latent profiles of holistic humanistic caring perception among newly graduated nurses; (2) examine demographic and occupational factors associated with profile membership; and (3) assess the indirect association between holistic humanistic caring perception profiles and clinical nursing competency through thriving at work.

## 2. Materials and Methods

### 2.1. Participants

A convenience sample of newly graduated nurses was recruited from six tertiary general hospitals in Shanghai and Taicang City between January and March 2026. The electronic questionnaire was administered via Wenjuanxing (wjx.cn), and all items were mandatory; consequently, there were no item‐level missing data. The questionnaire link was distributed to eligible newly graduated nurses through the participating hospitals. Based on pilot testing, a completion time of < 4 min was prespecified as a quality‐control criterion. Of the 454 submitted questionnaires, 17 were excluded because of completion times < 4 min and 16 because of internally inconsistent responses to a prespecified pair of semantically opposite items in the Thriving at Work Scale. Inconsistent responding was defined as selecting the same response option for both items. After these exclusions, 421 questionnaires (92.7%) were retained for analysis.

Inclusion criteria were as follows: (1) possession of a valid nurse practice certificate; (2) working experience in a healthcare institution of 2 years or less; and (3) completion of the orientation training for newly graduated nurses. Exclusion criteria were as follows: (1) individuals who were not members of the active nursing workforce at a participating hospital (e.g., nursing students or interns) and (2) absence from work for one consecutive month for any reason.

### 2.2. Sample Size

LPA generally requires a relatively large sample, and methodological guidance suggests that a sample of approximately 300 or more is preferable for obtaining stable profile solutions [24]. Allowing for a 20% nonresponse rate, the target sample size was therefore set at 375. Sensitivity analyses indicated that the final sample of 421 provided at least 80% power to detect a standardised indirect association of approximately 0.12 in the mediation analysis and odds ratios of ≥ 2.38 or ≤ 0.42 in the multinomial logistic regression [25, 26].

### 2.3. Ethical Statement

This study was approved by the Ethics Committee of the Medical College of Henan University (Approval No. HUSOM2026‐069).

## 3. Instruments

### 3.1. Self‐Designed Sociodemographic Characteristics Questionnaire

This questionnaire collected information on age, gender, educational level, only‐child status, professional title, marital status, nursing work experience (months), department, living situation, household registration type, employment status and average monthly income.

### 3.2. Holistic Humanistic Caring Perception Scale for Nurses

This scale was developed by Chen et al. [13]. It consists of 40 items across six dimensions: self‐care, care from hospital management, care from the department, care from patients and their families, care from family members and relatives and care from classmates, friends and peers. Each item is rated on a 5‐point Likert scale ranging from 1 (‘*never*’) to 5 (‘*always*’). The total score ranges from 40 to 200, with higher scores indicating a higher level of holistic humanistic caring perception. In this study, Cronbach’s *α* coefficient for the overall scale was 0.979.

### 3.3. Thriving at Work Scale

The scale was originally developed by Porath et al. [27] and translated into Chinese by Han et al. [28]. It comprises two dimensions (learning and vitality) with 10 items in total (five items for each dimension). Each item is scored on a 7‐point Likert scale ranging from 1 (‘*strongly disagree*’) to 7 (‘*strongly agree*’), with items 4 and 8 reverse‐scored. The total score ranges from 10 to 70, and a higher score reflects a higher level of thriving at work. This scale has been used in the Chinese nurse population with good reliability and validity [29]. In this study, Cronbach’s *α* coefficient for the overall scale was 0.867.

### 3.4. Competency Scale for Clinical Nurses

This scale was developed by Dai [8] to measure the competency of clinical nurses in general hospitals. It includes five dimensions (knowledge, professional skills, personal traits, motivation and social competence) with 28 items in total. Each item is rated on a 10‐point scale ranging from 1 (‘*completely inconsistent*’) to 10 (‘*completely consistent*’). The total score ranges from 28 to 280, with higher scores indicating higher clinical nursing competency. In this study, Cronbach’s *α* coefficient for the overall scale was 0.983.

### 3.5. Statistical Analysis

LPA was performed using Mplus 8.0, with the six dimension‐level mean scores of holistic humanistic caring perception entered as continuous indicators. Model fit was evaluated using the Akaike information criterion (AIC), Bayesian information criterion (BIC), sample‐size‐adjusted BIC (aBIC), entropy, Lo–Mendell–Rubin adjusted likelihood ratio test (LMR‐LRT) and bootstrap likelihood ratio test (BLRT) [30]. Lower AIC, BIC and aBIC values indicate better model fit, whereas entropy values closer to 1 indicate better classification quality. Significant LMR‐LRT and BLRT results indicate that a model with *k* profiles provides a better fit than a model with k−1 profiles. Classification agreement between the retained three‐profile solution and a five‐profile solution collapsed into corresponding low, moderate and high levels was additionally assessed using percentage agreement and Cohen’s *κ*. Descriptive analyses of participants’ demographic characteristics and study variables were conducted using SPSS 27.0. Categorical variables were presented as frequencies and percentages, and continuous variables as means ± standard deviations. Chi‐square tests were performed to identify factors associated with the LPA profiles, and significant factors were subsequently entered into a multinomial logistic regression analysis. Finally, mediation analysis was conducted using PROCESS macro Version 4.1 for SPSS (Model 4). Latent profile membership was specified as a multicategorical independent variable and indicator coding was applied, with Profile 1 (low humanistic caring perception) designated as the reference category [31]. Accordingly, two indicator variables were generated: the first compared Profile 2 (high humanistic caring perception) with Profile 1, and the second compared Profile 3 (moderate humanistic caring perception) with Profile 1. Thriving at work was entered as the mediator and clinical nursing competency as the outcome variable. To reduce potential confounding, age, work experience, monthly income, professional title, living situation, only‐child status, household registration type and employment status were included as covariates. Indirect effects were estimated using 5000 bootstrap samples and were considered statistically significant when the 95% bootstrap confidence interval did not include zero.

## 4. Results

### 4.1. Common Method Variance Test

Harman’s single‐factor test was used to assess potential common method bias. An unrotated exploratory factor analysis of all study items yielded eight factors with eigenvalues greater than 1, and the first factor accounted for 45.35% of the total variance. No single factor emerged or accounted for the majority of the variance, indicating no evidence of a single dominant common factor [32].

### 4.2. Latent Profiles of Holistic Humanistic Caring Perception

Using the six dimension‐level mean scores as indicators, one‐ to five‐profile models were estimated sequentially (Table 1). The AIC, BIC and aBIC decreased as the number of profiles increased. The three‐profile solution showed good classification quality (entropy = 0.892), with significant LMR‐LRT and BLRT results (both *p* < 0.001). The average posterior probabilities for the three profiles were 0.948, 0.971 and 0.932 respectively. The four‐profile solution had lower entropy (0.867), and its LMR‐LRT was not significant (*p* = 0.085). Although the five‐profile solution had the lowest information criteria, the highest entropy (0.969), significant LMR‐LRT (*p* = 0.009) and BLRT (*p* < 0.001), and a smallest class of 42 participants (10.0%), the unweighted averages of the six dimension means across the five ordered profiles were 2.58, 2.98, 3.45, 4.16 and 4.81 respectively. After collapsing the five‐profile solution into corresponding low, moderate and high levels, classification agreement with the retained three‐profile solution was 87.6% (Cohen’s *κ* = 0.814), with all disagreements occurring between adjacent levels. The dimension‐level mean patterns of the four‐ and five‐profile solutions are presented in Supporting Table S1; inspection of these patterns indicated that the additional profiles primarily represented finer gradations in overall caring levels rather than clearly distinct additional configurations. Considering the overall statistical evidence, parsimony and substantive interpretability, the three‐profile solution was retained.

**TABLE 1:** Latent model fitting indicators of newly graduated nurses’ holistic humanistic caring perception (*n* = 421).

Indices	1‐Profile	2‐Profile	3‐Profile	4‐Profile	5‐Profile
Fit statistics					
AIC	6988.921	5857.060	5539.447	5379.215	5257.751
BIC	7037.433	5933.870	5644.555	5512.622	5419.456
aBIC	6999.353	5873.577	5562.049	5407.903	5292.523
Entropy	—	0.922	0.892	0.867	0.969
LMR‐LRT	—	< 0.001	< 0.001	0.085	0.009
BLRT	—	< 0.001	< 0.001	< 0.001	< 0.001
Group size					
C1, *n* (%)	421 (100.0)	215 (51.1)	130 (30.9)	125 (29.7)	54 (12.8)
C2, *n* (%)		206 (48.9)	157 (37.3)	101 (24.0)	108 (25.7)
C3, *n* (%)			134 (31.8)	107 (25.4)	108 (25.7)
C4, *n* (%)				88 (20.9)	109 (25.9)
C5, *n* (%)					42 (10.0)

*Note:* aBIC, sample‐size‐adjusted Bayesian information criterion; LMR‐LRT, Lo–Mendell–Rubin adjusted likelihood ratio test. For the retained three‐profile solution, the average posterior probabilities for the most likely class membership were 0.948, 0.971 and 0.932 for Profiles 1, 2 and 3 respectively.

Abbreviations: AIC, Akaike information criterion; BIC, Bayesian information criterion; BLRT, bootstrap likelihood ratio test.

### 4.3. Participant Characteristics

Most participants were female (87.4%), aged 24–26 years (61.0%) and held a bachelor’s degree (72.9%). Further demographic and occupational characteristics are presented in (Table 2).

**TABLE 2:** Demographic and occupational characteristics of participants across latent profiles (*n* = 421).

Variables	Total sample, *n* (%)	1*n* (%)	2*n* (%)	3*n* (%)	χ^2^	*p*
Sex					1.929	0.381
Male	53 (12.6%)	12 (9.2%)	22 (14.0%)	19 (14.2%)		
Female	368 (87.4%)	118 (90.8%)	135 (86.0%)	115 (85.8%)		
Age group (years)					26.687	**< 0.001**
21–23	127 (30.2%)	29 (22.3%)	61 (38.9%)	37 (27.6%)		
24–26	257 (61.0%)	96 (73.8%)	73 (46.5%)	88 (65.7%)		
27–28	37 (8.8%)	5 (3.8%)	23 (14.6%)	9 (6.7%)		
Education level					7.548	0.110
Junior college or below	63 (15.0%)	20 (15.4%)	28 (17.8%)	15 (11.2%)		
Bachelor’s degree	307 (72.9%)	100 (76.9%)	111 (70.7%)	96 (71.6%)		
Master’s degree or above	51 (12.1%)	10 (7.7%)	18 (11.5%)	23 (17.2%)		
Marital status					1.219	0.543
Unmarried	397 (94.3%)	125 (96.2%)	147 (93.6%)	125 (93.3%)		
Married	24 (5.7%)	5 (3.8%)	10 (6.4%)	9 (6.7%)		
Only‐child status					31.325	**< 0.001**
Yes	126 (29.9%)	61 (46.9%)	26 (16.6%)	39 (29.1%)		
No	295 (70.1%)	69 (53.1%)	131 (83.4%)	95 (70.9%)		
Current living situation					31.250	**< 0.001**
Alone	197 (46.8%)	75 (57.7%)	56 (35.7%)	66 (49.3%)		
With partner	64 (15.2%)	8 (6.2%)	36 (22.9%)	20 (14.9%)		
With family	64 (15.2%)	10 (7.7%)	32 (20.4%)	22 (16.4%)		
Shared rental	96 (22.8%)	37 (28.5%)	33 (21.0%)	26 (19.4%)		
Household registration type					10.313	**0.006**
Urban	224 (53.2%)	54 (41.5%)	91 (58.0%)	79 (59.0%)		
Rural	197 (46.8%)	76 (58.5%)	66 (42.0%)	55 (41.0%)		
Nursing work experience (months)					48.546	**< 0.001**
0–6	93 (22.1%)	41 (31.5%)	26 (16.6%)	26 (19.4%)		
7–12	103 (24.5%)	44 (33.8%)	19 (12.1%)	40 (29.9%)		
13–18	118 (28.0%)	32 (24.6%)	52 (33.1%)	34 (25.4%)		
19–24	107 (25.4%)	13 (10.0%)	60 (38.2%)	34 (25.4%)		
Professional title					17.408	**< 0.001**
Nurse	315 (74.8%)	114 (87.7%)	105 (66.9%)	96 (71.6%)		
Senior nurse	106 (25.2%)	16 (12.3%)	52 (33.1%)	38 (28.4%)		
Average monthly income					30.585	**< 0.001**
< 6000	139 (33.0%)	51 (39.2%)	50 (31.8%)	38 (28.4%)		
6000–8000	203 (48.2%)	72 (55.4%)	60 (38.2%)	71 (53.0%)		
> 8000	79 (18.8%)	7 (5.4%)	47 (29.9%)	25 (18.7%)		
Employment status					20.081	**< 0.001**
Permanent staff (bianzhi)	19 (4.5%)	2 (1.5%)	14 (8.9%)	3 (2.2%)		
Directly contracted staff	147 (34.9%)	58 (44.6%)	40 (25.5%)	49 (36.6%)		
Agency‐employed staff	255 (60.6%)	70 (53.8%)	103 (65.6%)	82 (61.2%)		
Department					19.020	0.164
Internal medicine	125 (29.7%)	35 (26.9%)	50 (31.8%)	40 (29.9%)		
Surgery	110 (26.1%)	36 (27.7%)	41 (26.1%)	33 (24.6%)		
Obstetrics and gynaecology	6 (1.4%)	0 (0.0%)	3 (1.9%)	3 (2.2%)		
Paediatrics	26 (6.2%)	11 (8.5%)	8 (5.1%)	7 (5.2%)		
Emergency	48 (11.4%)	23 (17.7%)	15 (9.6%)	10 (7.5%)		
ICU	49 (11.6%)	13 (10.0%)	16 (10.2%)	20 (14.9%)		
Operating room	36 (8.6%)	6 (4.6%)	14 (8.9%)	16 (11.9%)		
Others	21 (5.0%)	6 (4.6%)	10 (6.4%)	5 (3.7%)		

*Note:* Bold values indicate statistically significant *p* values (*p* < 0.05).

### 4.4. Characteristics of the Latent Profiles of Holistic Humanistic Caring Perception

Figure 1 shows the six dimensions of holistic humanistic caring perception across the three profiles. The low HC profile (30.9%, *n* = 130) showed generally low scores, although caring from family and friends was relatively higher than work‐related caring. The high HC profile (37.3%, *n* = 157) showed the highest and relatively even scores across the six dimensions. The moderate HC profile (31.8%, *n* = 134) showed intermediate scores, with relatively lower caring from hospital management and the department.

**FIGURE 1:**
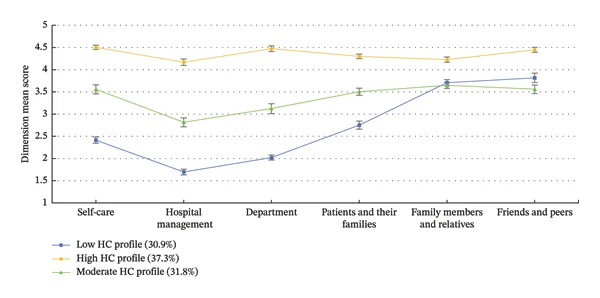
Latent profiles of holistic humanistic caring perception among newly graduated nurses. Note: Points represent estimated dimension‐level mean scores; error bars represent standard errors.

### 4.5. Factors Associated With Holistic Humanistic Caring Perception Profile Membership

The multinomial logistic regression model was statistically significant (*χ* ^2^(30) = 143.16, *p* < 0.001; Nagelkerke *R* ^2^ = 0.325). Significant profile‐specific associations were observed for only‐child status, living situation and work experience (Table 3).

**TABLE 3:** Multinomial logistic regression analysis of factors associated with holistic humanistic caring perception profile membership (*n* = 421).

Variables	Low HC vs. moderate HC				High HC vs. moderate HC			
	B	SE	Or (95% CI)	*p*	B	SE	Or (95% CI)	*p*
Only‐child status (ref: No)								
Yes	0.726	0.281	2.067 (1.193–3.581)	**0.010**	−0.775	0.310	0.461 (0.251–0.846)	**0.012**
Current living situation (ref: Shared rental)								
Alone	−0.255	0.335	0.775 (0.402–1.496)	0.448	−0.287	0.352	0.751 (0.377–1.495)	0.415
With partner	−0.437	0.581	0.646 (0.207–2.017)	0.452	0.005	0.495	1.005 (0.381–2.650)	0.992
With family	−1.099	0.492	0.333 (0.127–0.873)	**0.025**	0.101	0.410	1.106 (0.495–2.471)	0.806
Age group, years (ref: 27–28)								
21–23	−0.874	0.772	0.417 (0.092–1.894)	0.257	0.208	0.599	1.231 (0.380–3.984)	0.729
24–26	−0.133	0.703	0.876 (0.221–3.470)	0.850	−0.565	0.524	0.569 (0.204–1.588)	0.281
Professional title (ref: Senior nurse)								
Nurse	0.392	0.423	1.480 (0.646–3.392)	0.354	0.221	0.355	1.248 (0.622–2.504)	0.534
Average monthly income (ref: > 8000)								
< 6000	1.192	0.632	3.295 (0.956–11.363)	0.059	−0.135	0.490	0.873 (0.334–2.283)	0.783
6000–8000	0.974	0.575	2.648 (0.858–8.172)	0.090	−0.265	0.417	0.767 (0.339–1.736)	0.524
Nursing work experience, months (ref: 19–24)								
0–6	0.977	0.482	2.656 (1.032–6.833)	**0.043**	−0.706	0.412	0.494 (0.220–1.106)	0.086
7–12	0.564	0.450	1.759 (0.728–4.247)	0.210	−1.234	0.405	0.291 (0.132–0.643)	**0.002**
13–18	0.610	0.445	1.840 (0.769–4.402)	0.171	−0.037	0.352	0.963 (0.484–1.918)	0.915
Employment status (ref: Agency‐employed staff)								
Permanent staff (bianzhi)	0.859	1.070	2.361 (0.290–19.233)	0.422	1.093	0.749	2.983 (0.687–12.959)	0.145
Directly contracted hospital staff	0.324	0.273	1.383 (0.810–2.363)	0.235	−0.420	0.277	0.657 (0.382–1.131)	0.130
Household registration type (ref: Rural)								
Urban	−0.531	0.287	0.588 (0.335–1.031)	0.064	−0.047	0.275	0.954 (0.557–1.635)	0.865

*Note:* The moderate HC profile was the reference outcome category. Reference categories for categorical predictors are shown in parentheses. Bold values indicate statistically significant *p* values (*p* < 0.05).

Abbreviations: CI, confidence interval; OR, odds ratio.

### 4.6. Mediation Analysis of Thriving at Work

After adjustment for age, work experience, average monthly income, professional title, only‐child status, household registration type, living situation and employment status, both the high HC and moderate HC profiles were associated with higher thriving at work than the low HC profile (High vs. Low: *B* = 25.59, SE = 1.12, *t* = 22.84, *p* < 0.001; Moderate vs. Low: *B* = 15.11, SE = 1.07, *t* = 14.13, *p* < 0.001). Thriving at work was positively associated with clinical nursing competency (*B* = 1.80, SE = 0.16, *t* = 11.40, *p* < 0.001). The direct association remained significant for the high versus low comparison (*B* = 36.86, SE = 5.40, *t* = 6.83, *p* < 0.001), but not for the moderate versus low comparison (*B* = 7.97, SE = 4.16, *t* = 1.91, *p* = 0.056). Significant indirect associations through thriving at work were observed for both the high versus low comparison (*B* = 46.17, BootSE = 4.82, 95% bootstrap CI [36.73, 55.58]) and the moderate versus low comparison (*B* = 27.26, BootSE = 3.10, 95% bootstrap CI [21.30, 33.45]) (Table 4).

**TABLE 4:** Mediation analysis of thriving at work in the association between holistic humanistic caring perception profiles and clinical nursing competency (*n* = 421).

Effect	Profile comparison	Estimate	SE/BootSE	95% CI
Total effect	High HC vs. low HC	83.04	4.10	74.98–91.10
	Moderate HC vs. low HC	35.23	3.91	27.54–42.92

Direct effect	High HC vs. low HC	36.86	5.40	26.25–47.48
	Moderate HC vs. low HC	7.97	4.16	−0.22–16.15

Indirect effect through thriving at work	High HC vs. low HC	46.17	4.82	36.73–55.58
	Moderate HC vs. low HC	27.26	3.10	21.30–33.45

*Note:* The low HC profile was the reference category. Estimates are unstandardized coefficients. Models were adjusted for age, work experience, average monthly income, professional title, only‐child status, household registration type, living situation and employment status. Indirect effects were estimated using 5000 bootstrap samples. BootSE, bootstrap standard error.

Abbreviation: CI, confidence interval.

## 5. Discussion

This study identified three profiles of holistic humanistic caring perception that were distinguished mainly by their overall levels, with some variation in the relative distribution of caring sources. Caring from family and friends was relatively more prominent within the low HC profile, whereas caring from hospital management and the department was relatively less prominent within the moderate HC profile. For newly graduated nurses undergoing professional transition, relationships with family and friends generally predate entry into the workplace and may therefore provide relatively stable support, whereas supportive relationships with managers and colleagues need to develop within the clinical environment. When workplace support is still being established, nonwork social support may therefore become relatively more salient [17].

The association with only‐child status may partly reflect differences in prior experiences of support. Only children may receive relatively concentrated parental attention and family resources during childhood [33], which may shape their expectations of interpersonal caring after entering the workplace. During the transition to practice, differences between these expectations and the support encountered at work may influence perceptions of organisational caring. Previous research in China has also linked only‐child status with work adaptation among newly graduated nurses [34].

Living arrangements may also affect newly graduated nurses’ day‐to‐day access to nonwork support. Living with family may provide more regular companionship and more immediate emotional and practical support. By easing some of the practical and emotional demands outside work, such support may help nurses manage the transition to clinical practice [17, 35]. The greater accessibility of family support may therefore contribute to differences in perceived caring across living arrangements.

Work experience was also associated with profile membership, although the pattern across work‐experience groups was not linear. The first months of clinical practice are often marked by transition shock, unfamiliar clinical routines and the need to establish new workplace relationships, limiting opportunities to experience consistent caring and support at work [36, 37]. Nurses with longer work experience may be more familiar with routine clinical practice and have had more opportunities to establish stable workplace support. The association between work experience and caring profiles may therefore reflect differences across groups in clinical adaptation and access to workplace support rather than a simple increase in caring perception over time.

The association between caring profiles and clinical nursing competency may reflect differences in the support available to newly graduated nurses during the transition to independent practice. Caring within the workplace may provide timely guidance, feedback and practical assistance when difficulties arise [38, 39], while support from family and friends may ease some of the emotional and practical demands outside work [17, 35]. Self‐care may help newly graduated nurses regulate their own state, restore energy and maintain engagement at work. The acquisition and replenishment of these resources may help newly graduated nurses engage more effectively in clinical practice, thereby supporting the development of clinical nursing competency.

The indirect association between caring profiles and clinical nursing competency through thriving at work may be understood within conservation of resources theory [14]. The theory proposes that resource gains can facilitate further gains, as individuals with greater resources are better positioned to acquire additional resources [15]. For newly graduated nurses, greater caring‐related resources may provide a favourable basis for continued resource gain, which may be reflected at work in greater learning and vitality, the two components of thriving [18]. Continued learning may facilitate the acquisition and refinement of professional knowledge and clinical skills, whereas vitality may help nurses remain engaged when facing clinical demands and apply what they have learned in practice. Previous studies among nurses have similarly linked thriving at work with professional competence and job performance [19, 20]. Therefore, resources provided through caring may further support the development of clinical nursing competency by sustaining learning and vitality. This suggests that nurse managers may strengthen caring support while fostering work conditions that sustain learning and vitality, thereby supporting thriving at work and ultimately contributing to the development of clinical nursing competency among newly graduated nurses.

Notably, the two profile contrasts showed different patterns. The moderate‐versus‐low contrast was characterised primarily by an indirect association through thriving at work, whereas the high‐versus‐low contrast showed both indirect and direct associations with competency. This pattern suggests that thriving may be particularly relevant to competency differences between the moderate and low profiles, while other processes not captured in the present study may also be relevant to the difference between the high and low profiles.

Overall, these findings highlight the importance of tailoring caring support to nurses’ profile‐specific needs while creating conditions that sustain learning and vitality. For nurses in the low caring profile, caring from family and friends was relatively more prominent, suggesting that workplace caring may warrant particular attention. A consistent preceptor or mentor could provide continuity of support, while regular one‐to‐one communication may help identify difficulties in role adaptation and clinical practice. Timely recognition of progress, specific feedback and guidance when difficulties arise may provide both emotional and professional support during transition [39–41]. For nurses in the moderate profile, the relatively lower caring from hospital management and the department suggests that support should be strengthened at the organisational and unit levels. Nurse managers could recognise nurses’ progress, ensure access to necessary training and learning resources and regularly review workload and shift allocation to avoid excessive work demands during transition. These measures may help sustain learning and vitality [42, 43], while also strengthening nurses’ connection with the organisation and potentially supporting retention [44]. For nurses in the high caring profile, where caring was consistently favourable across sources, the emphasis may shift towards maintaining the existing caring environment and promoting continued development. Nurse managers could use periodic competency reviews to identify priorities for the next stage of development, encourage participation in case discussions, evidence‐based practice or unit‐based quality improvement activities and provide continuing education and professional development opportunities. As competence develops, greater autonomy and involvement in work decisions could be introduced accordingly [42, 43]. Taken together, profile‐specific management should address different caring needs while creating conditions that sustain learning, vitality and continued competency development.

## 6. Conclusions

This study identified three profiles of holistic humanistic caring perception among newly graduated nurses, characterised mainly by low, moderate and high overall levels, with some variation in the relative distribution of caring sources. Holistic humanistic caring perception profiles were associated with clinical nursing competency, with significant indirect associations through thriving at work. These findings may help nursing managers tailor support to different caring profiles, with attention to both thriving at work and the development of clinical nursing competency among newly graduated nurses.

### 6.1. Limitations

This study has several limitations. First, the cross‐sectional design does not permit conclusions about temporal ordering or causality; therefore, the observed indirect associations and differences across work‐experience groups should not be interpreted as evidence of causality or longitudinal change. Second, the use of self‐report measures at a single time point means that common method bias and social desirability bias cannot be completely excluded. Third, participants were recruited from six tertiary hospitals, which may limit the generalisability of the findings to other healthcare settings. Future longitudinal and multisource studies are warranted.

## Author Contributions

Yuping Zhang: conceptualisation, data curation, formal analysis and writing of the original manuscript. Xiaobing Yin and Guimei Wang: investigation, supervision and manuscript review. Dandan Shi and Weihua Hou: data collection and guidance on manuscript revision.

## Funding

This study was supported by the Shanghai Shenkang Hospital Development Center Municipal Hospital Diagnosis and Treatment Technology Promotion and Optimisation Management Project (Grant No. SHDC22023243).

## Disclosure

All authors reviewed and approved the final manuscript.

## Ethics Statement

This study adhered to ethical guidelines and complied with the principles of the Declaration of Helsinki. The study was approved by the Ethics Committee of the Medical College of Henan University (Approval No. HUSOM2026‐069). All participants provided informed consent before taking part in the survey.

## Consent

The authors have nothing to report.

## Conflicts of Interest

The authors declare no conflicts of interest.

## Supporting Information

Additional supporting information can be found online in the Supporting Information section.

## Supporting information


**Supporting Information 1** Supporting Table S1. Profile characteristics of the four‐ and five‐profile solutions based on dimension‐level means of holistic humanistic caring perception.

**Supporting Information 2** STROBE Statement—Checklist of items that should be included in reports of cross‐sectional studies.

## Data Availability

The data that support the findings of this study are available from the corresponding author upon reasonable request, subject to ethical restrictions and institutional policies.
