# Supplementary material for: The Relationship Between Holistic Humanistic Caring Perception, Thriving at Work and Clinical Nursing Competency Among Newly Graduated Nurses: A Mediation Study With Latent Profile Analysis
Source: J Nurs Manag. 2026 Oct 21;2026:1356643. doi: 10.1155/jonm/1356643 (PMC13686369; doi:10.1155/jonm/1356643)
Supplement: Supplementary file 1 [file JONM-2026-1356643-s001.docx]

**Supporting Table S1. Profile characteristics of the four- and five-profile solutions based on dimension-level means of holistic humanistic caring perception**

| **Solution** | **Profile** | **n (%)** | **Self-care** | **Hospital management** | **Department** | **Patients and their families** | **Family members and relatives** | **Friends and peers** |
| --- | --- | --- | --- | --- | --- | --- | --- | --- |
| Four-profile | C1 | 125 (29.7) | 3.308 | 2.563 | 2.807 | 3.324 | 3.533 | 3.467 |
|  | C2 | 101 (24.0) | 2.304 | 1.592 | 1.927 | 2.674 | 3.756 | 3.862 |
|  | C3 | 107 (25.4) | 4.100 | 3.564 | 3.900 | 3.931 | 3.988 | 4.088 |
|  | C4 | 88 (20.9) | 4.731 | 4.464 | 4.809 | 4.527 | 4.378 | 4.634 |
| Five-profile | C1 | 54 (12.8) | 2.232 | 1.149 | 1.735 | 2.634 | 3.697 | 4.009 |
|  | C2 | 108 (25.7) | 2.922 | 2.072 | 2.397 | 3.109 | 3.743 | 3.631 |
|  | C3 | 108 (25.7) | 3.589 | 3.014 | 3.304 | 3.517 | 3.640 | 3.656 |
|  | C4 | 109 (25.9) | 4.340 | 3.980 | 4.283 | 4.070 | 4.030 | 4.280 |
|  | C5 | 42 (10.0) | 4.800 | 4.920 | 4.930 | 4.770 | 4.700 | 4.770 |

Note. Values are model-estimated dimension-level means for each latent profile. n (%) represents participants assigned to each most likely profile. Class labels correspond to the original latent profile solutions and do not imply an ordinal ranking.
